# Medical Graduates and Students

**Published:** 1850-05

**Authors:** 


MEDICAL GRADUATES AND STUDENTS.

University of Pennsylvania, April 6, one hundred and sixty-nine.

Medical College of Georgia, March 5th, forty-four. Whole class one hundred and seventy-nine.

Rush Medical College, Feb. 7th, forty-four graduates.

Each of the Medical Schools of St. Louis had one hundred and ten students.

Louisville University, three hundred and seventy-six.

